# Hepatitis B virus reactivation in hepatocellular carcinoma patients after hepatic arterial infusion chemotherapy combined with and without immunotherapy

**DOI:** 10.1186/s13027-024-00574-7

**Published:** 2024-04-30

**Authors:** Lijie Zhang, Yiming Liu, Songlin Song, Joyman Makamure, Heshui Shi, Chuansheng Zheng, Bin Liang

**Affiliations:** 1 Department of Radiology, Union Hospital, Tongji Medical College, Huazhong University of Science and Technology, 1277 Jiefang Road, 430022 Wuhan, China; 2 Hubei Key Laboratory of Molecular Imaging, 430022 Wuhan, China

**Keywords:** Hepatic arterial infusion, Immunotherapy, Hepatocellular carcinoma, Hepatitis B virus, Reactivation

## Abstract

**Background:**

Hepatitis B virus (HBV) reactivation (HBVr) is a major concern for hepatocellular carcinoma (HCC) patients undergoing hepatic arterial infusion chemotherapy (HAIC) using mFOLFOX6 regimen. There is insufficient evidence to support the routine use of HAIC combined with immunotherapy in HCC patients with HBVr. The aim of this study was to examine the adverse events (AEs) related to HBVr in HCC patients after HAIC, with or without immunotherapy, and to assess the effectiveness of antiviral prophylaxis for HBVr.

**Methods:**

Medical records of HCC patients receiving HAIC combined with and without immunotherapy between January 2021 and June 2023 were reviewed. The patients were divided into two groups based on whether they received immunotherapy or not.

**Results:**

Out of the 106 patients, 32 (30.2%) developed HBVr. Among these, 23 eligible patients with HBVr were included, with 14 patients (61%) receiving immunotherapy and nine patients (39%) not receiving immunotherapy. Prior to HAIC treatment, four patients in each group had detectable HBV DNA with median titre of 3.66 × 10^2^ IU/ml (patients with immunotherapy) and 1.98 × 10^2^ IU/ml (patients without immunotherapy), respectively. Fifteen patients did not show detectable HBV DNA. At HBVr occurrence, the median HBV DNA level was 6.95 × 10^2^ IU/ml for all patients, 4.82 × 10^2^ IU/ml in patients receiving immunotherapy and 1.3 × 10^3^ IU/ml in patients not receiving immunotherapy. Grade 3 hepatitis developed in 12 cases of all patients (12/23, 48%), including five patients with immunotherapy (56%) and seven patients without immunotherapy (78%). At the 3-month follow-up, HBV DNA was detected in 10 patients, with a median HBV DNA level of 2.05 × 10^2^ IU/ml (range, 1.5 × 10^2^– 3.55 × 10^2^ IU/ml) in patients (7/10) with immunotherapy and 4.28 × 10^2^ IU/ml (range, 1.15 × 10^2^– 5.88 × 10^2^ IU/ml) in patients (3/10) without immunotherapy. Intensified antiviral treatment was administered to all patients. No HBVr-related fatal events occurred.

**Conclusion:**

HBVr can occur after HAIC combined with or without immunotherapy. The degree of liver damage did not differ significantly in patients treated with or without immunotherapy. Intensified antiviral treatment was found to be crucial for HCC patients with HBVr.

## Introduction

Liver cancer is a frequently occurring malignant tumor worldwide, with approximately 70% of new cases found in Asia alone [1]. The most prevalent form of liver tumor is hepatocellular carcinoma (HCC), which makes up 80% of cases and is typically observed in individuals with chronic liver diseases [2]. In Africa and East Asia, HCC is primarily caused by hepatitis B virus (HBV) infection, accounting for around 54% of cases [3]. Hepatic arterial infusion chemotherapy (HAIC) is a common treatment modality for advanced HCC, particularly in Asia [4].

The occurrence of HBV reactivation (HBVr) is a recognized complication of the HCC treatment [5]. While this complication is more common in patients who test positive for hepatitis B surface antigen (HBsAg) and antibody to hepatitis B core antigen (anti-HBc), it can also occur in individuals with resolved infections, as evidenced by negative HBsAg and positive anti-HBc [6]. HBVr can manifest in various clinical presentations, ranging from asymptomatic hepatitis to fatal liver damage [7]. Patients with HCC undergoing treatments such as systemic chemotherapy, immunotherapy and HAIC could experience HBVr [8–10]. To our knowledge, no documented cases of HBVr associated only with tyrosine kinase inhibitors (TKIs) in HCC treatment, such as sorafenib, apatinib, and lenvatinib, have been reported [5].

Immune checkpoint inhibitor (ICI) therapy has revolutionized the HCC treatment landscape [11–13]. However, the combination treatment of immunosuppressive agents and cytotoxic chemotherapy could also increase the risk of HBVr [14]. The mechanism remains incompletely understood. In the case of HAIC, the utilization of high doses of chemotherapy drugs is involved, which can result in the suppression of lymphocyte function and impact various pathways related to the immune system [15, 16]. This phenomenon may provide an explanation for the potential reactivation of HBV when HAIC is employed. In terms of immunotherapy, the induction of HBVr through ICI treatment might initially seem contradictory. The purpose of blocking inhibitory receptors is to enhance immune system function and suppress virus replication. However, in patients with chronic HBV infection, the virus often infects larger hepatocytes. Inhibit the immunosuppressive pathways could lead to the destruction of these hepatocytes and the release of previously dormant virus into circulation [17, 18]. Whereas, blocking PD-1 may promote T regulatory cells (T-regs) proliferation, thereby increasing immunosuppression and potentially leading to HBVr [19, 20].

The potential implications of HAIC combined with or without immunotherapy in patients with HBVr are not yet fully understood. Hence, the aim of this retrospective study is to investigate the safety of the combination therapy in HCC patients with HBVr.

## Materials and methods

### Study design and participants

A retrospective analysis was carried out on patients with HCC at our institution between January 2021 and June 2023. In this study, the requirement for written informed consent was waived, and prior approval from the Institutional Review Board was obtained before beginning the study.

The inclusion criteria for this study were as follows: (1) individuals over the age of 18 with advanced primary unresected HCC, confirmed through pathological or clinical diagnosis, and in accordance with the guidelines set forth by the American Association for the Study of Liver Diseases (AASLD) [21]; (2) patients solely treated with HAIC or a combination of HAIC and immunotherapy, without undergoing surgical operations or any other local treatments until reaching the primary endpoint during the study period; (3) individuals tested positive for HBV infection, specifically HBsAg, or tested negative for HBsAg but positive for anti-HBc; (4) patients diagnosed with HBVr based on the provided definition; (5) patients with regular monitoring of HBV DNA and liver function during hospitalization and follow-up, as recommended by the treating physician; (6) patients with presence of at least one measurable lesion; (7) patients with liver function classified as Child-Pugh Class A or B without ascites; and (8) an Eastern Cooperative Oncology Group (ECOG) performance status of 0–2.

Participants who met any of the following criteria were excluded from the study: co-infection with other hepatotropic viruses or HIV, lost to follow-up, obstructive jaundice, concurrent malignant comorbidities, serious non-malignant illnesses, history of hepatotoxic medication within eight weeks prior to HCC treatment [10], or a history of corticosteroid administration.

### HAIC treatment

The patients underwent a three-week cycle regimen, involving the intra-arterial insertion of a catheter into the hepatic artery, and a microcatheter into the feeding arteries of the tumor. The HAIC protocol was used for the administration of mFOLFOX6 treatment, which included infusion of oxaliplatin, calcium folinate and 5-fluorouracil (5-FU) (with a 10-minute interval after calcium folinate) on the first day at a dose of 85 mg/m^2^, 200 mg/m^2^ and 250 mg/m^2^ (I.V. for 15 min), respectively, followed by continuous arterial infusion of 5-FU for 46 h at a dose of 2,400 mg/m^2^.

Following the procedure, the catheter and sheath were removed and a pressure bandage was applied. The HAIC procedure was repeated until the tumor remained stable or progressed, or if intolerable toxicity was observed. Patients had the option to discontinue treatment if they declined the protocol. The HAIC treatment would only be initiated if the viral load decreased below the level of 10^5^ IU/ml.

### Immunotherapy

The immunotherapy drugs, tislelizumab, camrelizumab, sintilimab, and atezolizumab, were given to patients through IV infusion every three weeks, following the dose prescribed by the doctor. In case of disease progression or intolerable adverse events (AEs), the administration of immunotherapy was stopped. To manage symptoms, treatments like glucocorticoids or immune-suppressant agents were given, depending on the severity and the affected organs.

### Antiviral therapy

Based on the 2017 guidelines from the European Association for the Study of the Liver (EASL) [22], individuals diagnosed with chronic hepatitis B, either hepatitis B ‘e’ antigen (HBeAg) -positive or -negative, with HBV DNA levels exceeding 2,000 IU/ml, serum alanine aminotransferase (ALT) greater than the upper limit of normal (reference range less than 40 IU/L), and/or displaying moderate liver necroinflammation or fibrosis, should undergo antiviral therapy. Moreover, patients with compensated or decompensated cirrhosis requiring treatment should receive antiviral therapy regardless of their HBV DNA or ALT levels. If the patient was previously on antiviral therapy before hospitalization, the treatment was continued.

In our hospital, there were three types of oral antiviral drugs (nucleoside analogs) available: entecavir (ETV), tenofovir disoproxil fumarate (TDF), and tenofovir alafenamide fumarate (TAF).

To optimize the efficacy of antiviral treatment, patients who had previously received ETV or TDF before the onset of HBVr were additionally administered TAF, while those who had previously received TAF before the HBVr were additionally given ETV [23]. Concurrently, internal medicine treatment was administered. The treatment plan was modified as necessary until the HBV DNA became undetectable or the viral load decreased to the same level as before the HAIC.

### Definition

The primary endpoint was the hepatic AEs.

HBVr was defined as the initial detection of HBV DNA or a more than 10-fold increase (1 log10) in HBV DNA levels compared to the baseline value before HAIC treatment in individuals who have the HBsAg protein present. In the case of individuals who have cleared the infection (negative for HBsAg but positive for anti-HBc), reactivation is determined by the reversal of seroconversion to a positive HBsAg status [24].

Hepatitis was characterized by a threefold increase or more in the serum ALT levels, surpassing the reference range of 40 IU/L, or an absolute increase in ALT levels exceeding 100 IU/L. HBV-related hepatitis was defined as hepatitis occurring during or after HBVr, without any concurrent acute viral hepatitis infection or systemic disease. The classification of hepatitis was conducted by the treating physician and corresponding authors, considering clinical manifestations, laboratory tests, and imaging examinations. The categories included HBV-related, drug-induced, liver lesion progression-related, and immune-related hepatitis [25, 26].

The criteria for determining hepatic AEs were established based on the guidelines provided by the Common Terminology Criteria for AEs version 5.0 [27]. Grade 3 hepatitis was characterized by ALT levels greater than 5 times the upper limit of normal (ULN) to 20 times the ULN if the baseline levels were within normal range, or more than 5 to 20 times the baseline levels if the baseline levels were abnormal. Grade 4 hepatitis was defined as ALT levels exceeding 20 times the ULN if the baseline levels were within normal range, or more than 20 times the baseline levels if the baseline levels were abnormal.

Antiviral prophylaxis was described as the administration of antiviral treatment (nucleoside analogs) prior to and during HAIC treatment.

### Follow-up

Patients were monitored until the discontinuation of HAIC, occurrence of all-cause mortality, follow-up failure, or completion of the study, whichever came first.

The monitoring of patients comprised of physical examinations, laboratory assessments, and imaging procedures. Laboratory assessments, which encompassed liver function evaluations, screening for tumor markers, and tests for HBV (hepatitis B virus) (HBV serology and quantification of HBV DNA), were performed at three-week intervals during HAIC and every two months after therapy completion. Imaging procedures, specifically CT or MRI scans, were carried out every three months within the initial two years and every six months thereafter. Lung metastasis was confirmed through chest radiography conducted every six months. Additional imaging tests, such as chest CT, bone scintigraphy, and PET, were only conducted if clinically indicated.

### Statistical analysis

The demographic data, including mean, standard deviation, and percentage, were analyzed with the statistical software SPSS version 26.0. The data were presented in the form of means and standard deviations or medians and ranges. The comparison of categorical variables involved the utilization of the X^2^-test or Fisher’s exact test when appropriate, while the comparison of continuous variables utilized the Mann-Whitney U test or Wilcoxon Signed Rank test. Statistical significance was determined with a two-tailed *P* value of less than 0.05. Graphs were created using GraphPad Prism 8.0 (GraphPad Software Inc., San Diego, CA, USA).

## Results

### Background characteristics of patients with HBVr

**Fig. 1:**
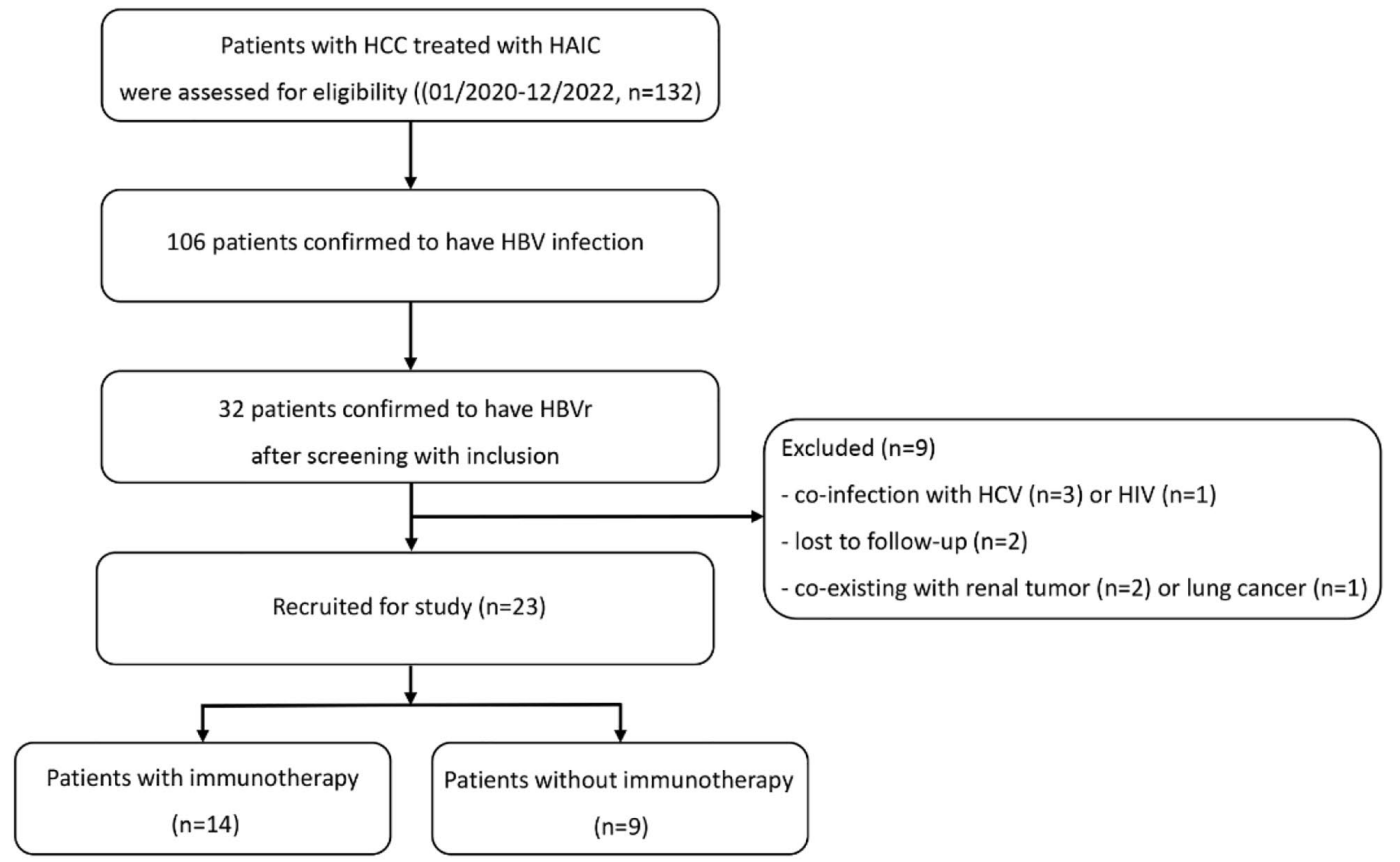
Flow chart depicting patient deposition. HCC, hepatocellular carcinoma; HAIC, hepatic arterial infusion chemotherapy; HBV, hepatitis B virus; HBVr, hepatitis B virus reactivation; HCV, hepatitis C virus; HIV, human immunodeficiency virus

This study screened a total of 132 patients who underwent HAIC treatment and confirmed that 106 patients had HBV infection. Among them, 32 patients (30.2%) were found to have HBVr, and 9 patients were excluded from the analysis. After applying rigorous inclusion and exclusion criteria, a total of 23 patients with HBVr were included in the study. Figure 1 illustrates the comprehensive experimental design, and Table 1 provides a summary of the patient characteristics. The majority of the study cohort consisted of male individuals (*n* = 18, 78%), with a median age of 56 years (range, 29–78 years). The patients received an average of 3.90 ± 2.208 HAIC cycles for HBVr treatment. At baseline, a detectable HBV DNA was found in 8 patients (35%), with a median titre of 3.2 × 10^2^ IU/ml (range, 1.32 × 10^2^– 7.31 × 10^3^ IU/ml). All patients received antiviral prophylaxis before undergoing HAIC therapy. Specifically, 12 patients were administered ETV, 2 patients received TDF, and 9 patients received TAF. Additionally, 20 patients tested positive for HBsAg, while 8 patients tested positive for serum HBeAg. Among the study participants, 19 patients were treated with TKIs, including Sorafenib (*n* = 6), Apatinib (*n* = 6), and Lenvatinib (*n* = 7). According to the Barcelona Clinic Liver Cancer (BCLC) staging system, the majority of patients fell into the BCLC-C category (*n* = 18, 78%). It is important to note that all tumor nodules observed in the patients were either multifocal or diffuse, although this information was not included in Table 1.

**Table 1:** Characteristics of enrolled patients

Variables	All patients (*n* = 23) ^*^	Patients with immunotherapy (*n* = 14) ^*^	Patients without immunotherapy (*n* = 9) ^*^	*P* value
Age (yrs)	58.26 ± 10.594	57.14 ± 13.755	54.22 ± 7.067	< 0.001
Sex				0.964
Male	18 (78%)	11 (79%)	7 (78%)	
Female	5 (22%)	3 (21%)	2 (22%)	
Number of HAIC cycles to reactivation	3.82 ± 2.208	4.07 ± 2.401	3.44 ± 1.944	< 0.001
HBsAg seropositivity	20 (87%)	12 (86%)	8 (89%)	0.824
HBeAg seropositivity	8 (34%)	5 (36%)	3 (33%)	0.906
HBcAb seropositivity	23 (100%)	14 (100%)	9 (100%)	-
Baseline HBV-DNA level				0.436
Undetectable	15 (65%)	10 (71%)	5 (56%)	
Detectable	8 (35%)	4 (29%)	4 (44%)	
Median baseline HBV DNA (range), IU/mL	0 (0–7310)	0 (0–448)	0 (0–7310)	
Type of NA				0.290
ETV	12 (52%)	8 (57%)	4 (44%)	
TDF	2 (9%)	2 (14%)	0 (0%)	
TAF	9 (39%)	4 (29%)	5 (56%)	
ALT (u/L)	33.78 ± 17.886	38.00 ± 21.318	27.22 ± 7.855	0.283
AST (u/L)	46.26 ± 23.224	54.14 ± 30.219	34.00 ± 8.109	0.147
TBIL (µml/L)	20.58 ± 9.237	18.30 ± 9.859	24.13 ± 7.307	0.035
Child-Pugh class				0.741
A	21 (91%)	13 (93%)	8 (89%)	
B	2 (9%)	1 (7%)	1 (11%)	
AFP (ng/mL)				0.265
< 100	12 (52%)	6 (43%)	6 (67%)	
≥ 100	11 (48%)	8 (57%)	3 (33%)	
Types of TKIs				0.824
None	4 (17%)	2 (14%)	2 (22%)	
Sorafanib	6 (26%)	3 (21%)	3 (34%)	
Apatinib	6 (26%)	4 (29%)	2 (22%)	
Lenvatinib	7 (31%)	5 (36%)	2 (22%)	
Types of ICIs				-
None	9 (38%)	-	9 (100%)	
Tislelizumab	3 (13%)	3 (21%)	-	
Camrelizumab	5 (22%)	5 (36%)	-	
Sintilimab	4 (17%)	4 (29%)	-	
Atezolizumab + Bevacizumab	1 (5%)	1 (7%)	-	
Sintilimab + Bevacizumab	1 (5%)	1 (7%)	-	
BCLC stage				0.279
B	5 (22%)	2 (14%)	3 (34%)	
C	18 (78%)	12 (86%)	6 (66%)	
Tumor size (cm)				0.800
≤ 5	4 (17%)	3 (21%)	1 (12%)	
5 ~ 10	10 (43%)	6 (43%)	4 (44%)	
≥ 10	9 (40%)	5 (36%)	4 (44%)	
ECOG Score				0.624
0	19 (83%)	12 (86%)	7 (78%)	
1	4 (17%)	2 (14%)	2 (22%)	

* Except where indicated, data are numbers of patients, with percentages in parentheses, or means ± standard deviations

HAIC, hepatic arterial infusion chemotherapy; HBsAg, hepatitis B surface antigen; HBeAg, hepatitis B ‘e’ antigen; HBcAb, hepatitis B virus core antibody; HBV, hepatitis B virus; NA, nucleoside analogs; ETV, entecavir; TDF, tenofovir disoproxil fumarate; TAF, tenofovir alafenamide fumarate; ALT, alanine aminotransferase; AST, aspartate transaminase; TBIL, total bilirubin; AFP, alpha fetoprotein; TKIs, tyrosine kinase inhibitors; ICIs, immune checkpoint inhibitors; BCLC, Barcelona Clinic Liver Cancer

The participants included in the study were divided into two subgroups depending on whether they underwent immunotherapy or not. A total of 14 individuals (61%) received immunotherapy, while nine individuals (39%) did not. The individuals who received immunotherapy were notably older (median age: 58.0 vs. 53.0 years old, *P* <.001) compared to those who did not. Moreover, there was a significant increase in the number of HAIC cycles to HBVr among participants who underwent immunotherapy (*P* <.001). With regards to laboratory analyses, individuals who underwent immunotherapy exhibited an elevation in aspartate aminotransferase (AST) levels and ALT, as well as a decrease in total bilirubin (TBIL) (*P* =.035) level, in comparison to individuals who did not receive immunotherapy. Among the cohort, the most commonly used immunotherapy drug was camrelizumab (*n* = 5, 22%), followed by sintilimab (*n* = 4, 17%) and tislelizumab (*n* = 3, 13%). There were no statistically significant differences observed for other variables in the two groups prior to the HAIC procedure.

### The treatment and outcome of HBVr and hepatitis

**Fig. 2:**
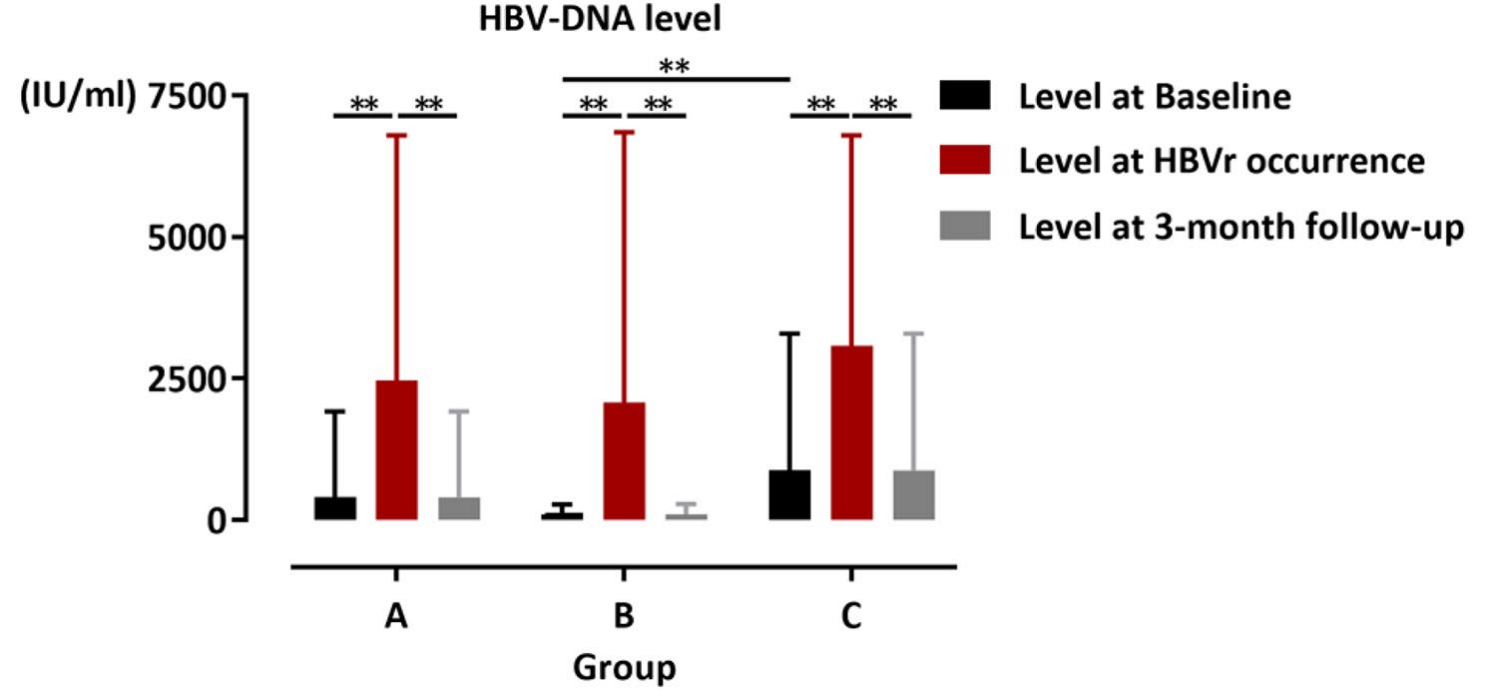
The HBV-DNA level at baseline and at HBVr occurrence. The significant increase in HBV-DNA levels was identified in all three cohorts at HBVr occurrence compared to baseline levels. No statistical difference of HBV-DNA level was observed between the Group B and Group C at HBVr occurrence and 3-month follow-up. Group A, the entire cohort; Group B, the patients with immunotherapy; Group C, the patients without immunotherapy. * *P* <.05; ** *P* <.001. HBV, hepatitis B virus; HBVr, hepatitis B virus reactivation

**Fig. 3:**
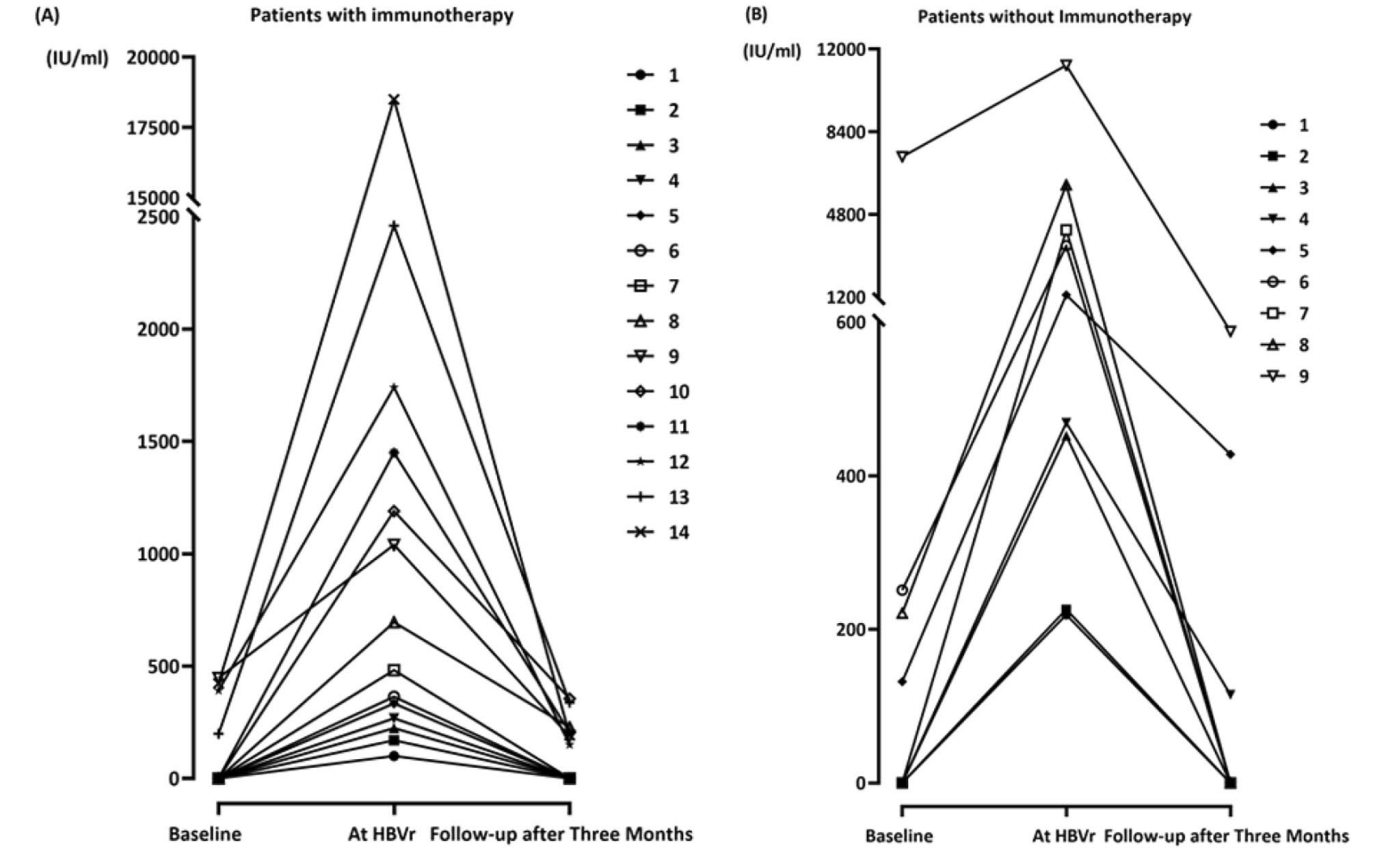
Kinetics of HBV DNA during treatment for each patient. (**A**) The changes of HBV DNA in patients with immunotherapy. (**B**) The changes of HBV DNA in patients without immunotherapy. HBV, hepatitis B virus; HBVr, hepatitis B virus reactivation

During the reactivation phase, the median level of HBV DNA for all patients was 6.95 × 10^2^ IU/ml (range, 1 × 10^2^– 1.85 × 10^4^ IU/ml). In patients undergoing immunotherapy, the median HBV DNA level was 4.82 × 10^2^ IU/ml (range, 1 × 10^2^– 1.85 × 10^4^ IU/ml), while in patients without immunotherapy, it was 1.3 × 10^3^ IU/ml (range, 2.19 × 10^2^– 1.13 × 10^4^ IU/ml). No statistical difference was observed between the patients with and without immunotherapy for HBV-DNA level when HBVr occurred. The study revealed a significant increase in HBV DNA levels in all three groups compared to the initial levels, regardless of whether immunotherapy was given. At a 3-month follow-up after HBVr occurrence, HBV DNA was not detected in 13 patients, including seven who were administered immunotherapy and six who were not. Among the remaining ten patients with detectable HBV DNA, seven cases were in the immunotherapy group and three patients were in the non-immunotherapy group. The median HBV DNA level in patients who received immunotherapy was 2.05 × 10^2^ IU/ml (range, 1.5 × 10^2^– 3.55 × 10^2^ IU/ml), while that was 4.28 × 10^2^ IU/ml (range, 1.15 × 10^2^– 5.88 × 10^2^ IU/ml) in patients without immunotherapy. However, no statistically significant difference was observed (Fig. 2). The changes of HBV DNA level in each patient are shown in Fig. 3.

**Fig. 4:**
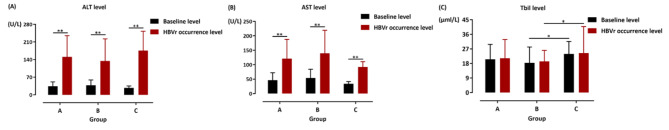
The changes of liver function test results at baseline and at HBVr occurrence. (**A**) The changes of ALT level in three cohorts. The significant increase in ALT levels at reactivation in all three cohorts compared to baseline levels was identified. A higher level was observed in group B compared to group C at baseline, while an increase in ALT level was identified in group C compared to group B at HBVr occurrence without statistical difference. (**B**) The AST level in all cohorts. A significant increase in AST levels at reactivation in all three cohorts compared to baseline levels was identified. The increasing trend of AST level was observed in group B compared with that in group C at baseline and at occurrence of HBVr without significant difference. (**C**) The TBIL level in three cohorts. An increase in TBIL level was observed in group C compared with that in group B at baseline and at occurrence of HBVr. Group A, the entire cohort; Group B, the patients with immunotherapy; Group C, the patients without immunotherapy. * *P* <.05; ** *P* <.001. HBVr, hepatitis B virus reactivation, ALT, alanine aminotransferase; AST, aspartate aminotransferase; TBIL, total bilirubin

Furthermore, all patients experienced an increase levels of ALT, AST, and TBIL when HBVr occurred after HAIC (Fig. 4). No significant difference was observed in ALT and AST when HBVr occurred. A significant increase for TBIL level in patients without immunotherapy compared with that in patients receiving immunotherapy was identified (*P* =.002). Fourteen patients who had previously received ETV or TDF before HBVr were administered TAF, while nine patients who had received TAF before HBVr were administered ETV. The present investigation revealed that among a group of 23 individuals, 12 patients experienced grade 3 hepatitis. Notably, patients who did not undergo immunotherapy exhibited a greater prevalence of this condition (78%, 7/9) compared to those who received immunotherapy (56%, 5/14) (Table 2). These results indicated a significant disparity in grade 3 hepatitis occurrence between the two treatment categories due to HBVr (*P* =.048) (Table 2). Fortunately, there were no fatalities attributable to liver failure within the patient cohort.

**Table 2:** Treatment-related AEs

Items		All patients (*n* = 23) ^*^	Patients with immunotherapy (*n* = 14) *	Patients without immunotherapy (*n* = 9) *	*P* Value
Total	Any	23 (100%)	14 (100%)	9 (100%)	-
	Grade ≥ 3	12 (52%)	4 (29%)	8 (89%)	0.004
HAIC attributed					
Abdominal pain	Any	20 (87%)	12 (86%)	8 (89%)	0.824
	Grade ≥ 3	0 (0%)	0 (0%)	0 (0%)	-
Fever	Any	21 (91%)	13 (93%)	8 (89%)	0.741
	Grade ≥ 3	0 (0%)	0 (0%)	0 (0%)	-
Nausea/vomiting	Any	19 (83%)	12 (86%)	7 (78%)	0.624
	Grade ≥ 3	0 (0%)	0 (0%)	0 (0%)	-
Transaminitis	Any	22 (96%)	13 (93%)	9 (100%)	0.412
	Grade ≥ 3	0 (0%)	0 (0%)	0 (0%)	-
HBVr attributed					
Hepatitis	Grade = 3	12 (52%)	5 (29%)	7 (78%)	0.048
	Grade = 4	0 (0%)	0 (0%)	0 (0%)	-
Systemic therapy attributed					
Secondary Hypertension	Any	8 (34%)	5 (36%)	3 (33%)	0.906
	Grade ≥ 3	0 (0%)	0 (0%)	0 (0%)	-
Protein urea	Any	3 (13%)	2 (14%)	1 (11%)	0.824
	Grade ≥ 3	1 (4%)	0 (0%)	1 (11%)	0.202
Hand-foot syndrome	Any	5 (22%)	3 (21%)	2 (22%)	0.964
	Grade ≥ 3	0 (0%)	0 (0%)	0 (0%)	-
Diarrhea/colitis	Any	3 (13%)	1 (7%)	2 (22%)	0.295
	Grade ≥ 3	0 (0%)	0 (0%)	0 (0%)	-
Rash	Any	3 (13%)	3 (21%)	0 (0%)	0.136
	Grade ≥ 3	0 (0%)	0 (0%)	0 (0%)	-
Fatigue	Any	6 (26%)	3 (21%)	3 (33%)	0.525
	Grade ≥ 3	0 (0%)	0 (0%)	0 (0%)	-
Constipation	Any	2 (9%)	2 (14%)	0 (0%)	0.235
	Grade ≥ 3	0 (0%)	0 (0%)	0 (0%)	-

* Except where indicated, data are numbers of patients, with percentages in parentheses, or means ± standard deviations

AEs, adverse events; HAIC, hepatic arterial infusion chemotherapy; HBVr, hepatitis B virus reactivation

### HAIC procedure and systemic therapy

During the study period, the entire patient cohort underwent a median of 13 cycles of HAIC, ranging from 6 to 20 cycles per patient. Among the patients who were administered immunotherapy, the median number of HAIC treatments was 12 cycles, with a range of 6 to 20 cycles. On the contrary, patients who did not receive immunotherapy had a median of 13 HAIC treatments, ranging from 9 to 16 cycles. The median duration of immunotherapy treatment was 16 weeks, ranging from 3 to 101 weeks. In total, seven patients experienced delays in HAIC or immunotherapy due to HBVr.

The research findings indicated that the occurrence of grade ≥ 3 adverse events was significantly higher in patients who did not receive immunotherapy (8/9, 89%), mainly attributable to HBVr-associated hepatitis (7/8, 78%). One patient experienced severe proteinuria due to the administration of TKI, which was relieved after reducing the dose of Lenvatinib (Table 2).

Additionally, a separate patient encountered catheter dislocation during HAIC treatment, necessitating catheterization to be repeated. However, there were no fatalities or discontinuations of HAIC procedures and immunotherapy due to complications.

## Discussion

Limited research exists on the impact of HAIC combined with or without immunotherapy on HBVr. This study identified a 30.2% incidence rate of HBVr in patients undergoing HAIC. Antiviral therapy was shown to reduce the severity of hepatitis in HBV-infected patients, even in those negative for HBsAg. Laboratory tests, including ALT, AST, and TBIL levels, indicated no significant difference in liver damage between patients receiving immunotherapy and those who did not, suggesting that immunotherapy did not greatly influence the development or severity of HBVr in these individuals.

HBVr can occur during immunosuppression in patients with chronic hepatitis B (anti-HBc-positive and HBsAg positive) or resolved infection (anti-HBc-positive and HBsAg negative) [28, 29]. Liu et al. [30] found that the occurrence of HBVr in HCC patients after HAIC was 11.7% (16/137) in the antiviral group and 27.3% (9/33) in the non-antiviral group. It is important to note that all patients included in that study were HBsAg-positive and did not receive immunotherapy. On the other hand, Zhang et al. [23] studied 114 cancer patients undergoing immunotherapy with HBsAg-positive status and observed that six patients (5.3%) developed HBVr. Yoo et al. [31] reported that among patients receiving immunotherapy for cancer treatment, the incidence rates of HBVr were 0.14% (5/3465) for all patients, 1.0% (5/511) for HBsAg-positive patients, and 0.0% (0/2954) for HBsAg-negative patients. These studies revealed different HBV serological patterns, including detectable HBV-DNA levels, HBsAg-positive status, HBeAg-positive status, and treatment regimen have an impact on the emergence of HBVr. Immunotherapy may have lower impact on HBVr than chemotherapy. In this study, an incidence rate of 30.2% was reported, which was higher than that reported in previous studies. On the one hand, that whether receiving antiviral treatment before HAIC and different HBV serological patterns was not considered as the grouping indicator due to the limit sample size of patients included in this study, which may also influence HBVr occurrence. On the other hand, our study was an observational retrospective analysis, and treatment biases may have been present. However, all cases included in this study received various types of antiviral drugs prior to HAIC. Therefore, the reliability of the main conclusion would not be impacted by whether preventive treatment was administered before HAIC.

The impact of antiviral therapy on the severity of HBVr-associated hepatitis has not been extensively studied, although it has been confirmed to prevent the emergence of HBVr. Zhang et al. [23] found a lower incidence of grade 3/4 hepatitis in patients who received antiviral prophylaxis compared to those who did not, although without statistical significance. Yoo et al. [31] reported no cases of HBV-associated hepatitis in the antiviral prophylaxis group, while two cases occurred in the non-antiviral prophylaxis group. However, it is important to note that all patients in these studies were HBsAg-positive cancer patients undergoing immunotherapy exclusively. In this research, antiviral drug therapy before HAIC treatment resulted in no grade 4 hepatitis or HBVr-related deaths. This suggests a potential protective effect of antiviral therapy on liver function, although further research is needed to explore the impact of different HBV serological patterns on the severity of HBVr-associated hepatitis. In addition, Lee et al. [32] conducted a review and follow-up of 62 consecutive patients with chronic hepatitis B or resolved HBV infection who underwent ICIs treatment for unresectable HCC. Their study concluded that a higher HBV viral load did not pose a contraindication for ICI treatment in HCC. Similarly, this research showed no instances of Grade 4 hepatitis attributed to HBVr in any patients, and there were no reported deaths due to liver failure. Therefore, based on previous studies and our own findings, it can be inferred that treatment with ICIs alone or in combination with HAIC is safe for HCC patients with HBsAg-negative and detectable HBV-DNA levels, as well as HBsAg-positive individuals. The combination treatment did not exacerbate impaired liver function.

The question of whether HCC patients with HBV undergoing locoregional therapies like transarterial chemoembolization (TACE), HAIC, and ablation should receive prophylactic antiviral drugs remains unresolved by the EASL guidelines 2017 [22] and AASLD guidelines 2016 [33] and 2018 [34]. HAIC treatment differs from TACE and ablation as it involves the use of large amounts of chemotherapy drugs, including 5-Fu, directly in the liver region [35]. This may have a similar impact on HBVr as systemic therapy to some extent. Viral replication can persist throughout a patient’s life, leading to chronic HBV infection with varying degrees of liver injury. Even when a ‘sero-virologic recovery’ occurs spontaneously, intra-hepatic covalently closed circular DNA (cccDNA) may persist and be responsible for HBV reactivation [5]. Therefore, all individuals undergoing chemotherapy and immunosuppressive therapy should be screened for HBsAg, anti-HBs and anti-HBc before starting immunosuppressive treatment. HBsAg-positive patients should receive ETV or TDF or TAF as treatment or prophylaxis [22]. In our study, all patients included had previous exposure to various antiviral drugs before HAIC, and the clinical benefits of antiviral therapy were confirmed. Therefore, considering the use of prophylactic antiviral drugs for cancer patients undergoing locoregional chemotherapy, especially those who are HBsAg-positive, could be beneficial. However, the optimal antiviral drug for cases of HBVr remains uncertain and requires further investigation.

This study has several limitations. Firstly, there was variability in the monitoring intervals of HBV DNA, both within and among patients, which may have underestimated the frequency and median duration of HBVr episodes. Secondly, the study lacked an ideal control group to investigate which prophylactic antiviral treatment provides the most long-term survival benefits. Lastly, the sample size was relatively small, limiting the exploration of various antiviral drugs and potential risk factors in preventing HBVr in patients receiving HAIC with and without immunotherapy.

## Conclusions

In conclusion, HBVr is a significant concern for patients with HCC undergoing HAIC. The administration of immunotherapy alongside HAIC does not affect the severity of HBVr in these patients, as compared to those who do not receive immunotherapy. Antiviral treatment plays a crucial role in managing HBVr. Further research is needed to investigate the efficacy of various prophylactic antiviral treatments in preventing HBVr and enhancing patient outcomes.

## Data Availability

Any further information can be requested from the corresponding author.

## Abbreviations

HBV: hepatitis B virus
HBVr: hepatitis B virus reactivation
HCC: hepatocellular carcinoma
HAIC: hepatic arterial infusion chemotherapy
HBsAg: hepatitis B surface antigen
anti-HBc: antibody to hepatitis B core antigen
TKIs: tyrosine kinase inhibitors
ICIs: Immune checkpoint inhibitors
T-regs: T regulatory cells
AASLD: American Association for the Study of the Liver Diseases
ECOG: Eastern Cooperative Oncology Group
5-FU: 5-fluorouracil
AEs: adverse events
EASL: European Association for the Study of the Liver
HBeAg: hepatitis B ‘e’ antigen
ETV: entecavir
TDF: tenofovir disoproxil fumarate
TAF: tenofovir alafenamide fumarate
ALT: alanine aminotransferase
ULN: upper limit of normal
AST: aspartate aminotransferase
TBIL: total bilirubin
TACE: transarterial chemoembolization
cccDNA: circular DNA
